# Spatial Properties of STDP in a Self-Learning Spiking Neural Network Enable Controlling a Mobile Robot

**DOI:** 10.3389/fnins.2020.00088

**Published:** 2020-02-26

**Authors:** Sergey A. Lobov, Alexey N. Mikhaylov, Maxim Shamshin, Valeri A. Makarov, Victor B. Kazantsev

**Affiliations:** ^1^Neurotechnology Department, Lobachevsky State University of Nizhny Novgorod, Nizhny Novgorod, Russia; ^2^Neuroscience and Cognitive Technology Laboratory, Center for Technologies in Robotics and Mechatronics Components, Innopolis University, Innopolis, Russia; ^3^Instituto de Matemática Interdisciplinar, Facultad de Ciencias Matemáticas, Universidad Complutense de Madrid, Madrid, Spain

**Keywords:** spiking neural networks, spike-timing-dependent plasticity, learning, neurorobotics, neuroanimat, synaptic competition, neural competition, memristive devices

## Abstract

Development of spiking neural networks (SNNs) controlling mobile robots is one of the modern challenges in computational neuroscience and artificial intelligence. Such networks, being replicas of biological ones, are expected to have a higher computational potential than traditional artificial neural networks (ANNs). The critical problem is in the design of robust learning algorithms aimed at building a “living computer” based on SNNs. Here, we propose a simple SNN equipped with a Hebbian rule in the form of spike-timing-dependent plasticity (STDP). The SNN implements associative learning by exploiting the spatial properties of STDP. We show that a LEGO robot controlled by the SNN can exhibit classical and operant conditioning. Competition of spike-conducting pathways in the SNN plays a fundamental role in establishing associations of neural connections. It replaces the irrelevant associations by new ones in response to a change in stimuli. Thus, the robot gets the ability to relearn when the environment changes. The proposed SNN and the stimulation protocol can be further enhanced and tested in developing neuronal cultures, and also admit the use of memristive devices for hardware implementation.

## Introduction

The adoption of brain-inspired spiking neural networks (SNNs) constitutes a relatively novel paradigm in neural computations with high potential, yet not fully discovered. One of the most intriguing and promising experimental illustrations of SNNs was the development of robots controlled by biological neurons, the so-called neuroanimates, proposed at the end of the XX century and currently attracting much attention (Meyer and Wilson, 1991; Potter et al., 1997; Reger et al., 2000; Izhikevich, 2002; Pamies et al., 2014; Dauth et al., 2016). In those experiments, neural networks self-organized in dissociated neuronal cultures, which was suggested to be used as a decision-making element in robotic systems. In the earlier 1990s, Meyer and Wilson introduced the term an animat, as a composition of words “animal” and “automat,” referring to a robot exhibiting the behavior of an animal (Meyer and Wilson, 1991). Later, several research groups developed prototypes of hybrid systems composed of a robot controlled by a living neural network. The main idea was to achieve adaptive learning in biological SNNs with a real physical embodiment.

Learning is inevitably linked with the interaction of an agent with its environment. Therefore, to implement learning *in vitro*, a neural network should be equipped with a “body” interacting with the environment. The first neuroanimat was proposed by Mussa-Ivaldi’s group (Reger et al., 2000). To control a tiny wheeled robot Khepera, they used electric potentials recorded from brain slices of the sea lamprey fed by signals from light sensors. Almost in parallel with this study, Potter et al. (1997) suggested connecting a neuronal culture grown on a multielectrode array (MEA) to animate a roving robot (DeMarse et al., 2001). They succeeded in constructing a virtual neuroanimat capable of moving in the desired direction within 60° corridor after 2 h of “training” with a success rate of 80% (Bakkum et al., 2008). Shahaf et al. (2008) used ultrasonic sensors detecting the presence of an obstacle in the trajectory of a neuroanimat by stimulating a neuronal culture, which, in turn, controlled the movement. Obstacles located on the right or left side provoked population bursts with different spiking signatures. Then, a computer algorithm detected and classified the population bursts and moved the robot in the corresponding direction.

Despite extensive experimental studies conducted over the last decades, the high computational potential of SNNs has not been really achieved. The main problem faced by the researchers building “living computers” is the absence of robust learning algorithms. Unlike the backpropagation algorithm (Rumelhart et al., 1986) and deep learning approaches (Lecun et al., 1998), which revolutionized artificial neural networks (ANNs), SNNs still lack similar methodology. In a more general context, the learning principles of biological neural networks are not explored up to the level sufficient for designing engineering solutions (Gorban et al., 2019). Several attempts were made to adapt the backpropagation algorithm and its variations to SNNs (Hong et al., 2010; Xu et al., 2013). Within this approach, an ANN is subject to learning, and then the obtained weights are transferred with some limitations to a similar SNN (Esser et al., 2016). However, SNNs trained in such a way usually do not achieve a level of accuracy similar to their ANN counterparts. This can be explained both by the formulation of the recognition problem and by the nature of the tests (Tavanaei et al., 2019).

One of the intriguing brain features is the ability to associative learning. It is based on synaptic plasticity, most likely of a Hebbian type (Hebb, 1949). A classic example of associative learning is Pavlovian conditioning (Pavlov, 1927). Generally, it binds a conditional stimulus (CS) with an unconditional stimulus (US). The US always evokes a response in the nervous system, whereas the CS initially does not. After several presentations of the US and CS together, the nervous system starts responding to the CS alone. Hebbian associative learning can be extremely efficient, given that the neural input dimension is high enough (Gorban et al., 2019; Tyukin et al., 2019). Experimentally, associative learning is often achieved in the form of operant or instrumental conditioning, which is characterized by the presentation of stimuli to an animal depending on its behavior (Pavlov, 1927; Hull, 1943; Dayan and Abbott, 2001).

There are several approaches to implement associative learning in mathematical models. One is to incorporate US and CS events as spiking waves or patches of activity propagating in neural tissue and associate them through a spatiotemporal interaction. Learning underlying such a “spatial computation” can be implemented by using spike-timing-dependent plasticity (STDP) (Gong and van Leeuwen, 2009; Palmer and Gong, 2014). The STDP implements the Hebbian rule. In this case, repeated arrival of presynaptic spikes a few milliseconds before the generation of postsynaptic action potentials leads to potentiation of the synapse, whereas the occurrence of presynaptic spikes after postsynaptic ones provokes synaptic depression (Markram et al., 1997; Bi and Poo, 1998; Sjöström et al., 2001). A different approach to the conditioning paradigm uses reinforcement learning, e.g. on the basis of an eligibility trace and dopamine modulated STDP (Houk et al., 1995; Izhikevich, 2007). Based on this type of plasticity, a robot interacting with humans capable of associating color and touch patterns was recently designed (Chou et al., 2015). However, this approach is quite complicated and was implemented only in model neural networks.

Many attempts to implement learning features in neuroanimats have been made in cultured neural networks grown *in vitro*. The use of synaptic plasticity as a mechanism of reinforcement or control of functional connections was demonstrated only in the case of relatively simple adaptive changes in the network. It has been suggested that the network homogeneity (e.g. unstructured connectivity) precludes the emergence of more complex forms of learning (Pimashkin et al., 2013, 2016). Earlier, we proposed an approach to explain the problems of learning in unstructured neural networks by the competition between different pathways conducting excitation to a neuron or set of neurons (Lobov S. A. et al., 2017; Lobov S. et al., 2017b). Recently, the possibility to structure the network geometry by directing axon growth was demonstrated experimentally (Malishev et al., 2015; Gladkov et al., 2017), which opens a new venue to build network architectures *in vitro*.

In this article, we study how spatial or topological properties of STDP can be used to implement associative learning in small SNNs. We show that the competition of spike-conducting pathways in a network plays an essential role in establishing the association of neural connections. In particular, on the network scale, STDP potentiates the shortest neural pathways and depresses alternative longer pathways. It permits replacing irrelevant associations by new ones in response to changes in the structure of external stimuli. We show that a roving robot controlled by an especially designed SNN can exhibit classical and operant conditioning. Application of the shortest-pathway rule allows the robot to relearn sensory-motor skills by rewiring the SNN on the fly when the environment changes. The developed SNN topology and the stimulation protocol can be adapted further for structured neural network cultured *in vitro* and for designing hardware SNNs based on, e.g. memristive plasticity.

## Materials and Methods

### The SNN Model

To simulate the dynamics of a SNN, we adopt the approach described elsewhere (Lobov S. A. et al., 2017). Briefly, the dynamics of a single neuron is given by Izhikevich (2003):

(1) $$\frac{d\,v}{d\,t}=0.04\,v^{2}+5\,v+140-u+I\,\left(t\right),$$

(2) $$\frac{d\,u}{d\,t}=a\,\left(b\,v-u\right),$$

where *v* is the membrane potential, *u* is the recovery variable, and *I*(*t*) is the external driving current. If *v* ≥ 30, then *v* ← *c*, *u* ← *u* + *d*, which corresponds to generation of a spike. We set *a* = 0.02, *b* = 0.2, *c* = −65, and *d* = 8. Then, the neuron is silent in the absence of the external drive and generates regular spikes under a constant stimulus, which is a typical behavior of cortical neurons (Izhikevich, 2003, 2004). The driving current is given by:

(3) $$I\,\left(t\right)=\xi \,\left(t\right)+I_{s\,y\,n}\,\left(t\right)+I_{s\,t\,m\,l}\,\left(t\right),$$

where *ξ*(*t*) is an uncorrelated zero-mean white Gaussian noise with variance *D*, *I*_*syn*_(*t*) is the synaptic current, and *I*_*stml*_(*t*) is the external stimulus. As a stimulus, we use a sequence of square electric pulses of the duration of 3 ms delivered at 10 Hz rate, with the amplitude sufficient to excite the neuron.

The synaptic current is the weighted sum of all synaptic inputs to the neuron:

(4) $$I_{s\,y\,n}\,\left(t\right)=\sum_{j}g_{j}\,w_{j}\,\left(t\right)\,y_{j}\,\left(t\right),$$

where the sum is taken over all presynaptic neurons, *w*_*j*_ is the strength of the synaptic coupling directed from neuron*j*, *g*_*j*_ is the scaling factor, in this paper we set them equal to 20 or -20 (Lobov S. A. et al., 2017) for excitatory and inhibitory neurons, respectively, and *y*_*j*_(*t*) describes the amount of neurotransmitters released by presynaptic neuron *j*.

To model the neurotransmitters, we use Tsodyks-Markram’s model (Tsodyks et al., 1998) that accounts for short-term depression and facilitation. We use this model with the following parameters: the decay constant of postsynaptic currents τ_*I*_ = 10 ms, the recovery time from synaptic depression τ_*rec*_ = 50 ms, the time constant for facilitation τ_*facil*_ = 1 s.

The dynamics of the synaptic weight *w*_*ij*_ of coupling from an excitatory presynaptic neurons *j* to a postsynaptic neuron *i* is governed by the STDP with two local variables (Song et al., 2000; Morrison et al., 2008). Assuming that τ_*ij*_ is the time delay of spike transmission between neurons *j* and *i*, a presynaptic spike fired at time *t*_*j*_ and arriving to neuron *i* at *t*_*j*_ + τ_*ij*_ induces a weight decrease proportional to the value of the postsynaptic trace *s*_*i*_. Similarly, a postsynaptic spike at *t*_*i*_ induces a weight potentiation proportional to the value of the presynaptic trace *s*_*j*_. The weighting functions obey the multiplicative updating rule (Song et al., 2000; Morrison et al., 2008). Thus, the weight dynamics is given by:

(5) $$\frac{d\,s_{i}}{d\,t}=-\frac{s_{i}}{\tau_{S}}+\sum_{t_{i}}\delta \,\left(t-t_{i}\right),$$

(6) $$\frac{d\,s_{j}}{d\,t}=-\frac{s_{j}}{\tau_{S}}+\sum_{t_{j}}\delta \,\left(t-t_{j}-\tau_{i\,j}\right),$$

(7) $$\frac{d\,w_{i\,j}}{d\,t}=\lambda \,\left[\left(1-w_{i\,j}\right)\,s_{j}\,\delta \,\left(t-t_{i}\right)-\alpha \,w_{i\,j}\,s_{i}\,\delta \,\left(t-t_{j}-\tau_{i\,j}\right)\right],$$

where τ_*S*_ = 10 ms is the time constant of spiking traces, λ = 0.001 is the learning rate, and α = 5 is the asymmetry parameter.

We implemented the SNN model (see below) as custom software NeuroNet developed in QT C++ environment. For the axonal delays, we used τ_*ij*_ = 3 ms for parallel connections and τ_*ij*_ = 4.2 ms for diagonal coupling. The selected delays are proportional to the interneuron distances and thus take into account the network topology. The app supports SNNs with up to 10^4^ neurons. On an Intel^®^ Core^TM^ i3 processor, the simulation can be performed in real time for a SNN with tens of neurons.

### Mobile Robot and Unconditional Motor Response

**FIGURE 1:**
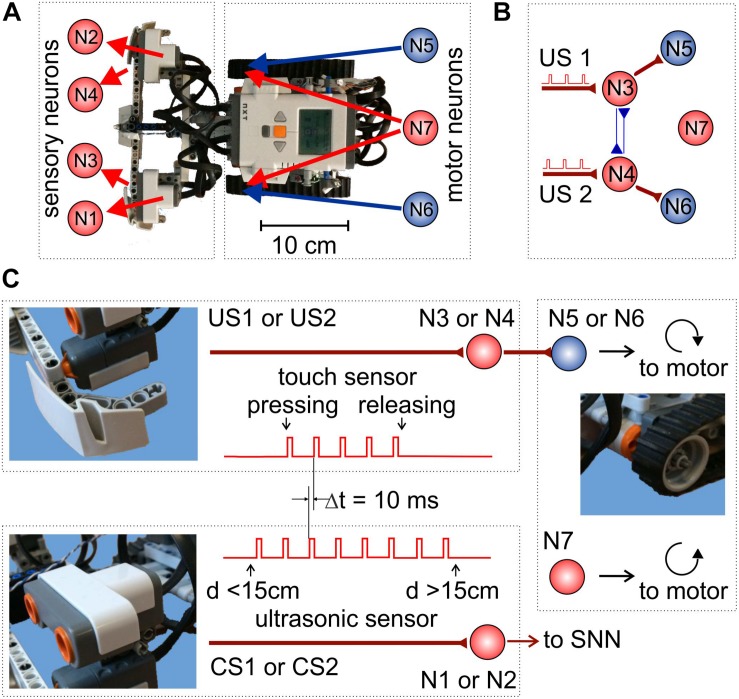
Experimental setup. **(A)** Mapping of the sensory and motoneurons in the mobile LEGO robot. **(B)** Simple SNN controlling basic robot movements and providing unconditional responses to touch stimuli. **(C)** Signaling pathways. Touch (top) and sonar (bottom) sensory neurons receive stimulating trains of rectangular pulses from the corresponding sensors. Then, motoneurons drive the robot’s motors.

We built a robotic platform from a LEGO^®^ NXT Mindstorms^®^ kit. Figure 1A shows the mapping of the robot sensors and motors to the sensory- and motoneurons, respectively. NeuroNet software was used to implement SNNs of different types controlling the robot behavior. Figure 1B illustrates the simplest SNN providing the robot with unconditional responses to touching events (see below). The software was run on a standalone PC connected to the robot controller through a Bluetooth interface.

The robot is equipped with two touch sensors and two ultrasonic sonars (Figure 1C). A sensitive bumper detects touch stimuli (collisions with obstacles) from the left and right side of the robot (Figure 1B). When a touch sensor is on, the corresponding sensory neuron (either N3 or N4) is stimulated by a train of pulses delivered at 10 Hz rate (Figure 1C, top-left panel). Such stimulation models signal processing in the sensory system of animals. The ultrasonic sonars are located above the bumper and are coupled to sensory neurons N1 and N2 (Figure 1C, bottom-left panel). A sonar sensor turns on if the distance to an obstacle is less than 15 cm. Then, the corresponding neuron is stimulated by a train of square pulses delivered at 10 Hz rate.

The SNN controls the robot movements through the activation of motoneurons. Motor neuron N7 produces tonic spiking with the mean frequency *F*, which is mapped simultaneously to the left and right motors. As a result, the robot moves straightforward with the velocity proportional to *F*. Neurons N5 and N6 are coupled to the right and left motors, respectively. The amount of neurotransmitters released by these neurons modulates the rotation velocity of the corresponding motor. When N5 (N6) fires, the right (left) motor slows down (or even rotates backward if, e.g. *F* = 0), and the robot turns to the right (left).

The robot also has three LEDs facilitating its recognition in the arena by a zenithal video camera. Video frames, captured at 29 Hz rate, were analyzed offline. Trajectory tracking was performed by employing a computer vision algorithm implemented in the OpenCV library. Robot detection is based on the fact that the robot image is a high gradient area. The LEDs turn off when a touch sensor is activated, which allows such events to be detected by analyzing the overall glow of the robot image.

The touch sensors mediate US (Figures 1B,C, top). When one of them is activated due to a collision with an obstacle, the corresponding sensory neuron (N3 or N4) starts firing and directly excites a motoneuron (N5 or N6, Figure 1B). As a result, the corresponding motor starts rotating backward, and the robot turns away from the obstacle and thus avoids the negative stimulus (Supplementary Video S1).

The sonars are connected to sensory neurons N1 and N2 and mediate CS. At the beginning of learning, the CS in the form of an approaching obstacle does not evoke any robot’s response. The goal of learning is to associate CS with US to avoid the obstacles in advance without touching them. To provide stimulation of “sensory neurons”, according to the STDP protocol, the stimulating pulses from the touch sensors have a 10-ms delay relative to the sonar pulses (Figure 1C).

## Results

### The Shortest Pathway Rule

**FIGURE 2:**
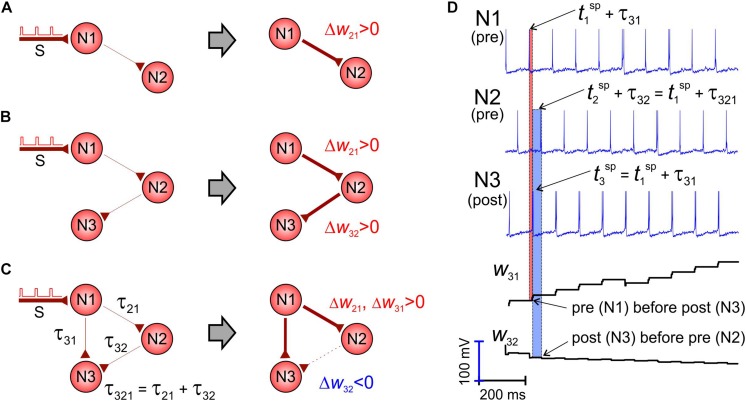
The shortest pathway rule. STDP potentiates the shortest pathways and inhibits alternative connections (W_*ij*_, τ_*i*__*j*_ are the weight and axonal delay of the coupling from neuron *j* to neuron *i*). **(A,B)** *Left*: Initial situation. *Right*: After STDP. The link width corresponds to the synaptic strength. Presynaptic spikes in a unidirectional chain precede postsynaptic spikes and STDP potentiates synaptic couplings. **(C)** The shortcut from neuron N1 to N3 makes the coupling from N2 to N3 “unnecessary” and STDP depresses it. **(D)** Spikes in the network and evolution of synaptic weights.

Let us consider a pair of unidirectionally coupled neurons driven by periodic stimuli applied to one of them (Figure 2A). Stimuli excite the first neuron, and then the activation propagates along the “chain” to the second cell, which fires, given that the coupling strength *w*_*21*_ is strong enough. Then, the presynaptic spikes precede the postsynaptic ones, and, as a result, the weight increases following the STDP rule (the first term in the right-hand side of Eq. 7). Such a situation can be extended into a chain of three or even more neurons (Figure 2B). Thus, STDP increases the corresponding synaptic weights.

However, if we add a new connection from the first neuron to the third one (Figure 2C), the weight dynamics changes crucially. Although all synapses are excitatory, the coupling directed from the second to the third neuron is depressed, while the other two are potentiated. This occurs because the axonal delay via the direct way N1–N3 (τ_31_, Figure 2C) is significantly shorter than the delay via the pathway N1–N2–N3 (τ_321_ = τ_21_ + τ_32_, Figure 2C). Thus, the first neuron makes fire directly the third one (which is also postsynaptic for *w*_*32*_), and its spikes appear ahead of the spikes coming from the second neuron (presynaptic for *w*_*32*_). Such an inverse sequence (Figure 2D) forces depression of the coupling *w*_*32*_ according to the STDP rule (the second term in the right-hand side of Eq. 7). We thus can formulate the shortest pathway rule:

• On the network scale, STDP potentiates the shortest neural pathways and depresses alternative longer pathways.

### SNN Exhibiting Non-trivial Associative Learning

**FIGURE 3:**
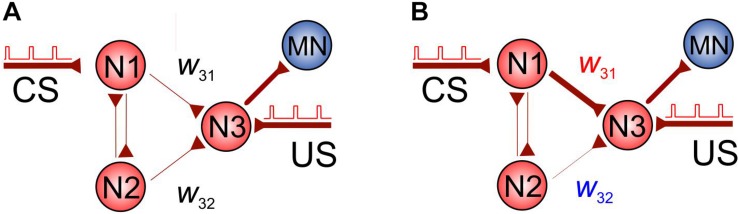
Associative learning based on the spatial properties of STDP. **(A)** The initial SNN. **(B)** Potentiation of the coupling *w*_*31*_ and depression of *w*_*32*_ during simultaneous stimulation of neuron N3 and N1 (US pulses are applied with a delay of 10 ms relative to CS pulses in order to comply with the STDP protocol).

Let us now employ the shortest-pathway rule to implement conditional learning in an SNN. Figure 3A shows a simple SNN consisting of four neurons, which can exhibit associative learning. The SNN receives two types of inputs: CS and US applied to neurons N1 and N3, respectively. To comply with the STDP protocol of paired stimulation, we assume that the US pulses arrive with a delay of 10 ms relative to CS pulses (see also Figure 1C).

At the beginning, the coupling between N1 and N3, *w*_*31*_, is not sufficient to excite N3 through the CS pathway. However, under stimulation, it is potentiated due to the appropriate delay between US and CS. At the same time, the coupling between N2 and N3, *w*_*32*_, is depressed due to the shortest pathway rule. Thus, after learning, we get the network shown in Figure 3B and the CS alone can activate neuron N3 and then the motoneuron. We also note that, similarly, if the CS is applied to N2 instead of N1, then *w*_*32*_ will be potentiated, while *w*_*31*_ depressed, and we get the same effect of associative learning.

### SNN Driving Robot

**FIGURE 4:**
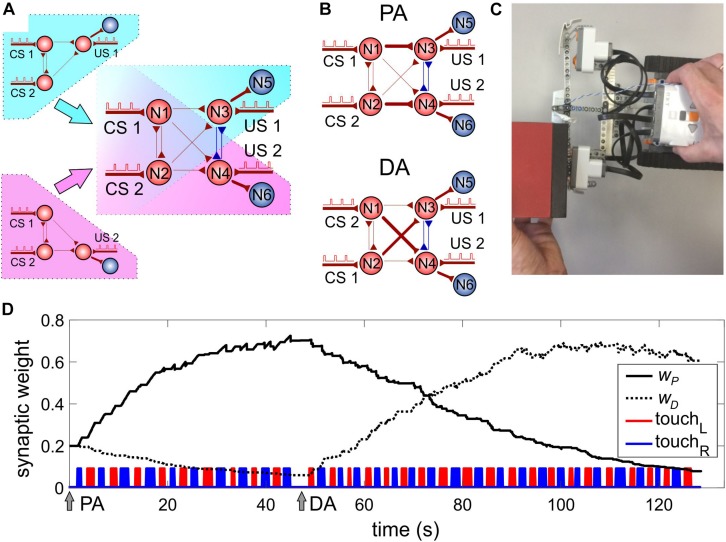
Model of classical conditioning. **(A)** The design of a two-channel SNN by duplicating the single-channel SNN (Figure 3) sharing some neurons. The neural circuit includes neurons N1–N4 involved in learning. Motoneurons N5 and N6 provide turning the robot away from an obstacle. **(B)** The SNN after learning. PA, parallel association: N1 (N2) is associated with N3 (N4), couplings *w*_*31*_ and *w*_*42*_ are potentiated. DA, diagonal association: N2 (N1) is associated with N3 (N4), couplings *w*_*32*_ and *w*_*41*_ are potentiated. **(C)** Application of a stimulus to the touch and sonar sensors. **(D)** Evolution of the average weights of parallel (*w*_*P*_) and diagonal (*w*_*D*_) couplings under classical conditioning. Arrows PA and DA denote the time instants of the beginning of learning with correspondent scheme of the US mapping; touchL (touchR) is the time course of triggering the left (right) touch sensor.

The above-discussed SNN (Figure 3) has one motoneuron and hence can drive one motor channel. To process events coming from the right and left sensors of the robot, we need to extend the SNN to account for two motor channels. Thus, we duplicate the SNN shown in Figure 3 but, at the same time, share some of the neurons between two copies of the SNN (Figure 4A). The resulting SNN contains four sensory neurons (N1, N2 for CS and N3, N4 for US, Figure 4A) and two motoneurons N5, N6 modulating the rotation velocities of the left and right motors, respectively (see also Figure 1). Neurons N3 and N4 are mutually inhibitory coupled with fixed synaptic weights (*w*_34_ = *w*_43_ = 1).

The pair of neurons receiving CS (N1, N2) can be connected to the pair of sonars in an arbitrary order (left–right or right–left). Depending on the connection, there can be two types of associations between the stimuli and motors: either with strong “parallel” (PA) or strong “diagonal” (DA) pathways (Figure 4B). Such freedom ensures that there is no *a priori* chosen structure in the complete SNN. Instead, the SNN adapts to the stimuli coming from the environment. Thus, the mutual exchange of the CS sources can simulate a situation with a change in the environment, which should induce relearning in the SNN and adaptation to novel conditions. Note that the bidirectional coupling between neurons N1 and N2 plays a fundamental role by providing synaptic competition while training couplings to neurons N3 and N4.

### Classical or Pavlovian Conditioning

To implement Pavlovian (classical) conditioning, let us, for a moment, deactivate neuron N7 responsible for forward movement. If an object approaches the robot from one side, the corresponding touch sensor is activated, and we get an unconditional response (Figure 4C and Supplementary Video S1). At the same time, the corresponding sonar is also triggered on, and paired trains of stimuli innervate sensory neurons with a time delay of 10 ms.

We repeated such a stimulation alternately on the left and right sides of the robot. This protocol led to the potentiation of two associations for the left and right sides. Five stimulating cycles applied to the right and left sides were sufficient to achieve robust learning. After switching the connections of the sonars between sensory neurons N1 and N2, the SNN was able to relearn the associations (i.e. to switch between PA and DA, Figure 4B) after about 10–15 stimulus cycles.

In practice, to avoid obstacles successfully, the robot should gain high selectivity of the right and left channels. Then, in the presence of an obstacle on the left side, neuron N5 fires while neuron N6 is silent, which occurs in part due to inhibitory connections between neurons N3 and N4. Experimentally, the channel selectivity can be monitored by measuring the ratio of synaptic weights of “parallel” and “diagonal” connections:

(8) $$w_{P}=\left(w_{31}+w_{42}\right)/2,w_{D}=\left(w_{41}+w_{32}\right)/2.$$

Figure 4D shows the dynamics of these connections when simulating classical conditioning. Note that in the case of PA, the parallel connection *w*_*P*_ is potentiated, while the diagonal connection *w*_*D*_ is depressed. This happens due to simultaneous potentiation/depression of the pairs (*w*_31_,*w*_42_) and (*w*_41_,*w*_32_), according to the shortest pathway rule. After switching the CS inputs (Figure 4D, DA arrow), the opposite effect is observed, which leads to relearning in the SNN.

To achieve a high learning rate, our experiments show that the SNN should satisfy the following conditions:

1. Intermediate noise variance (*D* = 5.5 in experiments).
2. Bidirectional coupling between CS neurons (N1 and N2, Figure 4A).
3. Couplings between CS and US neurons are STDP-driven.
4. Inhibitory connections between US neurons (N3 and N4, Figure 4A).

Condition (1) agrees with our previous findings showing that the network rearrangement under stimulation takes place in a certain interval of the noise intensity (Lobov S. A. et al., 2017). At low noise intensity, the neuronal activation may not reach the level necessary for STDP-ordered pre and post-synaptic spiking. At high noise intensity, random STDP events dominate and break learning (see Supplementary Figure S1). Condition (2) expresses competition between the synapses involved in the associations increasing the SNN selectivity. Thus, competition plays a positive role in learning, unlike the case study reported previously (Lobov S. et al., 2017b). Condition (3) implies a reduction of the SNN selectivity due to a negative effect that STDP can have on the synaptic couplings between CS neurons (*w*_*21*_ and *w*_*12*_). Condition (4) leads to competition between neurons “for the right” to be activated and, as a result, to an increase in the selectivity of the connections of the right and left channels.

### Operant or Instrumental Conditioning

**FIGURE 5:**
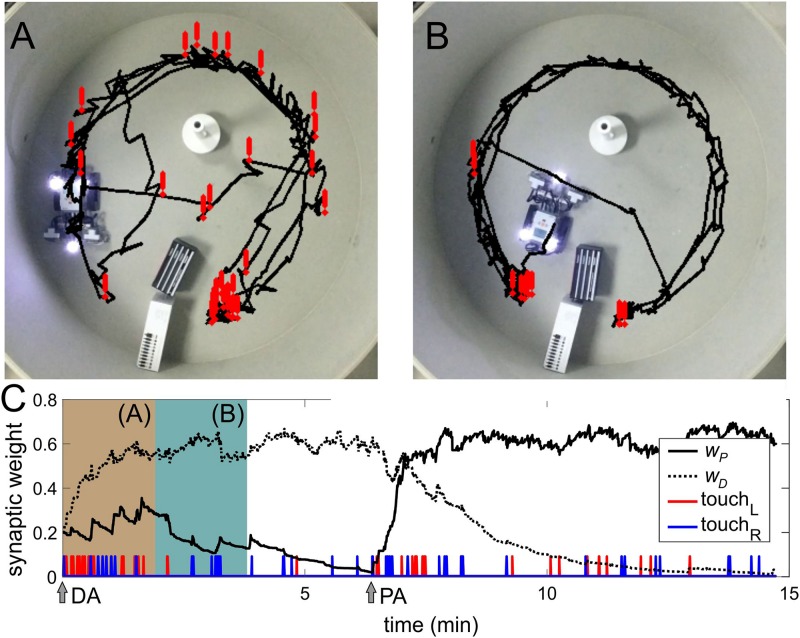
Operant conditioning. **(A)** Trajectory of the robot in the first 2 min of the experiment. Exclamation marks indicate the positions of collisions with obstacles. **(B)** Same as in **(A)** but after learning. **(C)** Evolution of the weights of parallel (*w*_*P*_) and diagonal (*w*_*D*_) couplings (compare to Figure 4D). Beige and green-blue bars correspond to periods **(A,B)**, respectively.

Animals learn behaviors through active interaction with the environment. To model such natural learning, we use operant (or instrumental) conditioning. To implement it, we activated motoneuron N7 (Figures 1B,C) responsible for forward movement and introduced the robot in an arena with several obstacles (Figure 5A).

In the beginning, the robot could avoid obstacles only after touching them due to US (Figure 5A). Then, learning progressively established associations between approaching obstacles (sonars, CS) and touching events (US). Thus, the robot learned to avoid obstacles in advance, without touching them (Figure 5B and Supplementary Videos S2, S3). We then switched sonars. Similarly to classical conditioning, the robot was able to relearn the associations (Figure 5C, PA arrow).

The learning rate depends on the total time of activation of the touch sensors. In turn, this time depends on the configuration of the arena, i.e. the arena size and the number of obstacles. In the Morris water maze (Figure 5A, 1 m^2^), learning takes about 2 min. In a larger room (50 m^2^) with a few obstacles, the learning time increases to 10–20 min. Relearning takes about twice a longer time.

In the operant conditioning, the SNN selectivity did not reach the value achieved in classical conditioning (compare Figures 4D, 5C). It occurs due to the fact that in the arena, the robot can approach objects in front. In this case, both sonars detect them, which leads to a simultaneous generation of stimuli on the left and right sides and competition between two connections from the same sensory neuron. Technical constraints, such as a narrow sensing angle of the sonars, also affect the correct implementation of the obstacle-avoidance task negatively. All these factors diminish the learning quality. Therefore, the robot sometimes collides with obstacles. Thus, in a real environment, learning does not reach 100% collision avoidance.

## Discussion

Competition is a universal paradigm well-extended both in neurophysiology, e.g. in the form of lateral inhibition (Kandel et al., 2000) and the ANN studies, e.g. in the form of competitive learning in Kohonen networks (Kohonen, 1982) or imitation learning (Calvo Tapia et al., 2018). In this work, we have proposed an SNN model implementing associative learning through an STDP protocol and temporal coding of sensory stimuli. To achieve successful learning, the SNN makes use of two mechanisms of competition. The first type is neuronal competition, i.e. different neurons compete to be the first to get excited. In our case, this mechanism was provided by inhibitory connections between US neurons.

The second type of mechanism is synaptic competition; i.e. different synaptic inputs to a single neuron compete to be the one exciting the neuron. This mechanism has been less addressed in the literature on learning. Earlier, it was shown that in unstructured networks, synaptic competition leads to negative consequences for learning (Lobov S. A. et al., 2017; Lobov S. et al., 2017b). We have shown that the proposed structured architecture of the SNN, together with synaptic competition implementing the STDP-mediated rule of the shortest pathway, can ensure learning. We also note that the proposed mechanism of synaptic competition works well in the case of temporal coding of stimuli. Stimulus coding by the firing rate may require the development of a different approach. For example, in our recent study (Lobov et al., 2020), we implemented synaptic competition using synaptic forgetting, depending on the activity of the postsynaptic neuron. This allowed performing a mixed type of coding (temporal and rate) in the problem of recognition of electromyographic signals.

To test the SNN, we used it for controlling a mobile robot. We have shown that indeed, the robot exhibits successful learning at the behavioral level in the form of classical and operant conditioning. During navigation in an arena, the SNN self-organizes in such a way that after learning, the robot avoids obstacles without collisions, relying on CS only. Moreover, it can also relearn if the connection of CS sensors is switched between the corresponding sensory neurons, and a network rewiring, widely observed in biological neural networks, is required (Calvo Tapia et al., 2020). The mechanism of relearning can be considered as a model of the animals’ ability to adapt to changes in the environment. In the SNN, it is possible due to synaptic competition. Our experiments have also shown that learning is robust. The robot can operate in environments of different sizes and with varying densities of obstacles.

The proposed SNN implements a model with two associations: left and right sensors “coupled” to the right and left turns. In general, such associative learning can be extended to multiple inputs and outputs. Thus, the proposed architecture can be considered as a perceptron composed of spiking neurons with two inputs and two outputs, where logical 1 or 0 at an input corresponds to the presence or absence of a CS, respectively. Then, the US provides a learning mechanism on how to excite the target neuron in the output layer, i.e. how to obtain the desired output. Thus, we get a simple mechanism for supervised learning, i.e. a replacement of the backpropagation algorithm for SNNs. However, the question of how many neurons such a spiking perceptron can contain and, hence, how many classes can be discriminated in this way requires additional studies.

We note that the parameters of sensory stimuli play a crucial role in the learning of behaviors. For example, longer delays between stimuli or their inverse order (CS after US) can impair learning. In this sense, the temporal coding in SNNs requires fine-tuning of the neuronal circuits and maybe not robust. The rate coding using, e.g. the triplet-based STDP rule (Pfister and Gerstner, 2006), voltage-based STDP with homeostasis (Clopath et al., 2010), or STDP together with BCM rule (Wade et al., 2008; Liu et al., 2019) is likely to increase the reliability of robot control. However, in this case, we may end up with a mixed type of coding (temporal and rate).

Due to structural simplicity, the proposed SNN and the learning algorithm admit a hardware implementation by, e.g. using memristors, which are adaptive circuit elements with memory. Memristors change their resistance depending on the history of electrical stimulation (Wang et al., 2019). Since the first experiments and simulations (Linares-Barranco et al., 2011), significant progress has been achieved in the implementation of excitatory and inhibitory STDP by using resistive-switching devices (RRAM), which are a particular class of memristors with two-terminal metal–insulator–metal structure. Although most of STDP demonstrations still rely on a time overlap of pre- and postsynaptic spikes (Yu et al., 2011; Kuzum et al., 2013; Emelyanov et al., 2019), the rich internal dynamics of higher-order memristive devices related to multi-time-scale microscopic transport phenomena provides timing- and frequency-dependent plasticity in response to non-overlapping input signals in a biorealistic fashion (Du et al., 2015; Kim et al., 2015). Memristive plasticity can be realized at different time scales, in particular with STDP windows of the order of microseconds (Kim et al., 2015), which is essential for the development of fast spike encoding systems.

Upon reaching the technology maturity, arrays of memristive synapses offer unique scalability being integrated with CMOS layers and showing spatiotemporal functions (Wang W. et al., 2018), as well as combined with artificial memristive neurons (Wang Z. et al., 2018) within a single network. Simple spiking architectures of Pavlov’s dog association have been proposed on memristors (Ziegler et al., 2012; Milo et al., 2017; Tan et al., 2017; Minnekhanov et al., 2019). However, more sophisticated architectures are required to reproduce different types of associative learning to be adopted in advanced robotic systems. We anticipate that, soon, artificial neurons can be realized on the CMOS architecture, whereas the STDP can be implemented by incorporating memristors (Emelyanov et al., 2019). It seems convenient to have paired micro-scaled memristive devices to reproduce bipolar synaptic weights. They can be mounted in a standard package for easier integration into the SNN circuits.

Finally, we also foresee that the provided architecture can be implemented in biological neural networks grown in neuronal cultures *in vitro.* Modern technology of microfluidic channels permits building different network architectures (Gladkov et al., 2017). On the one hand, such a living SNN could verify if our understanding of the learning mechanism at the cell level is correct. From the other side, biological neurons have a much higher level of flexibility mediated by different molecular mechanisms that may shed light on how learning and sensory-motor control are organized in nature.

## Data Availability Statement

The datasets generated for this study are available on request to the corresponding author.

## Author Contributions

SL, VM, and VK conceived and designed the research. SL developed the NeuroNet software, designed the robot configuration, and implemented the control of the robot by SNN. SL and MS carried out experiments with the robot and video tracking the movements of the robot. AM suggested the approach for emulating synaptic plasticity by memristive microdevices. All authors participated in the interpretation of the results and wrote the manuscript.

## Conflict of Interest

The authors declare that the research was conducted in the absence of any commercial or financial relationships that could be construed as a potential conflict of interest.
