# Supplementary material for: Spatial Properties of STDP in a Self-Learning Spiking Neural Network Enable Controlling a Mobile Robot
Source: Front Neurosci. 2020 Feb 26;14:88. doi: 10.3389/fnins.2020.00088 (PMC7054464; doi:10.3389/fnins.2020.00088)
Supplement: Supplementary file 1 [file Data_Sheet_1.pdf]

Supplementary Material

Supplementary Video

S1 (Classical conditioning):
https://drive.google.com/file/d/1StjtnQGOMkLiHwVi667grPIN_GLFsgvh/view

S2 (Before operant learning):
https://drive.google.com/file/d/1AaMxyDBqF3qitvcxMIO25-U8rlRto4Pe/view

S3 (After operant learning):
https://drive.google.com/file/d/1gY6nK2eFuvvUXuU3YIdJ0rE4TP5FUHs_/view

Supplementary Figure

The *sFigure* illustrates a "neuronal triangle" used for studying the influence of neural noise on learning (see also Fig. 3 in the main text). If we first stimulate neuron N1 by stimulus CS1, we expect potentiation of weight w_{31}. Then, if we stimulate neuron N2, STDP potentiates w_{32}, and depresses w_{31} due to the shortest pathway rule (N2-N1-N3 is a long path). For proper circuit functioning, time-to-time spikes must pass through the N1-N2 coupling. sFigure B shows that this requirement is met under moderate noise only. Weak noise leads to interruption of communication between neurons N1 and N2, whereas strong noise causes random spiking, not associated with stimulation of the neighboring neuron. As a result (sFig. C, D), we observe that under weak noise, both w_{31} and w_{32} are potentiated. Thus, the SNN "remembers" all the influences and cannot be retrained. Intermediate noise enables the SNN retraining. One of the connections is potentiated, while the other is depressed (the order depends on the exposure times T1 and T2). Under strong noise, STDP does not potentiate significantly both connections, which leads to learning disruption.

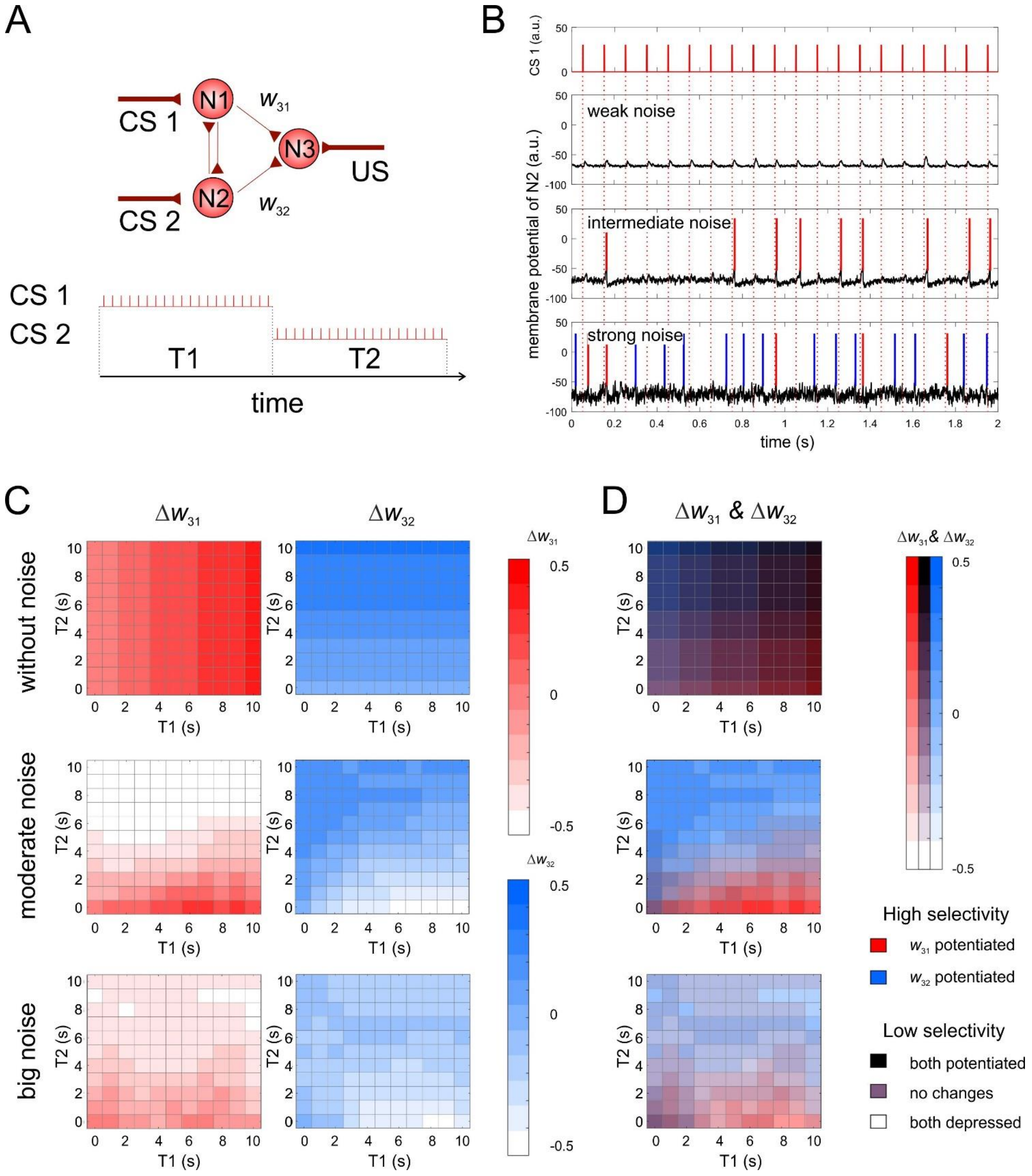

sFigure. Influence of noise on the learning quality. A) The scheme of simulation involving sequential stimulation of neuron N1 during time interval T1, and neuron N2 during time interval T2. B) Example of dynamics of neuron N2 during stimulation of neuron N1 under different noise intensity: "weak noise" D = 1, "intermediate noise" D = 5.5, "strong noise" D = 14. Spikes induced by stimuli are marked by red color, random spikes are in blue. C) Weight changes (separate for w_{31} and w_{32}) after simulation. D) Combination of the changes in the weights.
